# 

**Published:** 1890-01-25

**Authors:** 


### THE HOSPITAL

A WEEKLY JOURNAL OF
Science, Medicine, Nursing & Philanthropy.

SATURDAY, JANUARY 25th, 1890.

**The Extension of the Pension Fund** - 255
**Words of Consolation**—The Infancy of Christ - 256
**The Doctor's Pulpit.**—In Self-Defence - 257
**Popular Pastimes as Aids to Hospital Funds.**—Demonstrations. By A. W. WEBSTER - 258
**The Third-Class Carriage** - 259
**Robert Browning's Views of Life and Death** - 260
**Everybody's Page** - 261
**Notes and News** - 262
**Annotations.**—Lead Poisoning in Sheffield—The Irish Lunacy Service - 264
**The Practitioner's Outlook and Retrospect:**
MEDICINE—Cause of Typhoid Fever—Fœtal Malaria - 265
SURGERY—Ovariotomy and Listerism—Treatment of Carbuncle and Boils, 265; Cancer - 266
DIETETICS—Food for Invalids - 267
**Hospital Out-Patient Reform and Provident Dispensaries.** By ROBERT R. RENTOUL, M.D. - 267
**Cottage Nurses and Their Training.**—An Abstract of Miss Broadwood's Paper - 268
**Fallow Crops.**—The Hospital Saturday Fund Awards - 270
**Scraps and Gleanings** - 270

**EXTRA SUPPLEMENT—THE NURSING MIRROR.**

**En Passant.**—The Exhibition at Liverpool—Lay Nurses in Paris—Sisterhoods and Influenza—Thousands of Pounds—Cottage Hospitals and the Pension Fund—Kemerton Nursing Association—Reductio ad Absurdum - lxix
**Mr. Trantum's Hobby**—Concluded - lxx
**The Nurses' Bookshelf**—Obstetric Nursing - lxxi
**A Visit to Winchester** - lxxi
**Appointments—Death in Our Ranks** - lxxii
**Notices—Vacancies** - lxxii
**Amusements and Relaxation** - lxxii

